# Integrative Multi-Omics and Machine Learning Unveil Plakophilin 1 as a Robust Diagnostic Biomarker for Thymoma

**DOI:** 10.3390/biomedicines14102151

**Published:** 2026-09-23

**Authors:** Yunhan Li, Shen Zhang, Qian Gao, Shanshan Ding, Mengling Sun, Haiying Xue, Fan Yang, Yangyang Liu, Xiaokang An, Xiaotao Dong, Guoyu Zhang

**Affiliations:** 1 Department of Thoracic Surgery, The First Affiliated Hospital, Henan University, Kaifeng 475004, China; 2 Laboratory of Receptor Gene Regulation and Drug Discovery, School of Basic Medical Sciences, Henan University, Kaifeng 475004, China; 3 Kaifeng Key Laboratory of Translational Medicine for Thoracic Diseases, Kaifeng 475004, China

**Keywords:** thymoma, multi-omics, biomarker, PKP1, machine learning, single-cell RNA sequencing, virtual knockout simulations

## Abstract

**Background**: Thymoma (THYM) is a rare epithelial tumor originating in the anterior mediastinum. Due to the lack of specific clinical manifestations and reliable molecular biomarkers, its diagnosis and treatment are often delayed until advanced stages. Although multi-omics technologies have provided new strategies for biomarker discovery, systematic integrative studies specifically focused on thymoma remain limited. **Objectives**: This study aimed to identify and validate potential diagnostic biomarkers for thymoma through the integration of multi-omics data and machine learning approaches. **Methods**: We integrated transcriptomic, proteomic, and epigenomic datasets and applied machine learning algorithms to screen for core candidate genes. The cell-type-specific expression patterns of the identified candidates were further evaluated using single-cell RNA sequencing (scRNA-seq), and their potential molecular functions were predicted via in silico perturbation analysis. In addition, immunohistochemistry (IHC) was performed to validate the differential expression of the candidate biomarker at the protein level. **Results**: Six core candidate genes—interferon regulatory factor 6 (IRF6), thyroid hormone receptor interactor 6 (TRIP6), plakophilin 1 (PKP1), cadherin 1 (CDH1), pre-B-cell leukemia transcription factor 1 (PBX1), and keratin 1 (KRT1)—were identified through machine learning. Among these, PKP1 demonstrated excellent diagnostic performance in both the training set (area under the receiver operating characteristic (ROC) curve, AUC: 0.83; sensitivity: 0.80; specificity: 0.875) and the validation set (AUC: 0.94; sensitivity: 0.93; specificity: 1.000). Single-cell RNA sequencing confirmed a significantly enriched expression pattern of PKP1 in thymoma tumor cells. In silico perturbation analysis suggested that PKP1 may be involved in cell adhesion and tumor progression through regulation of the extracellular matrix (ECM)–integrin–cytoskeleton axis. Immunohistochemistry further validated the significantly elevated protein expression level of PKP1 in thymoma tissues. **Conclusions**: This study identifies PKP1 as a potential diagnostic biomarker for thymoma with high diagnostic performance and tumor cell-enriched expression, providing new insights into the molecular mechanisms underlying thymoma progression and its potential clinical applications.

## 1. Introduction

Thymomas are rare malignancies arising from thymic epithelial cells [1], accounting for only 0.2–1.5% of all malignant tumors, yet representing the most common tumor type in the anterior mediastinum, accounting for approximately 47% of cases [2]. The annual incidence of thymomas ranges from 1.3 to 3.2 cases per million individuals [2,3,4]. The precise etiology of thymomas remains unclear, with increasing age being the most widely recognized risk factor to date [5,6]. Although ionizing radiation may be associated with disease development, the available evidence remains insufficient [7]. According to the World Health Organization (WHO) classification, thymomas are primarily categorized into the following subtypes: type A, type AB, type B1, type B2, and type B3 [8]. Thymoma subtypes are not associated with sex (*p* = 0.389) but are associated with age. B2/B3 thymomas are more frequently observed in patients younger than 30 years, AB thymomas are more common among individuals aged 40–60 years, B1 thymomas are more prevalent in those aged 50–60 years, whereas type A and micronodular thymomas are predominantly observed in individuals older than 70 years, with micronodular thymoma showing the highest incidence in this age group [9]. Approximately 30% of patients are asymptomatic and are frequently diagnosed incidentally. Symptomatic patients commonly present with cough and chest pain, which are primarily attributable to a local mass effect [10]. Approximately 30% of patients also have concomitant myasthenia gravis (MG) [11].

Similar to most malignancies, the treatment of thymoma generally requires a multimodal therapeutic approach [12]. Currently, surgical resection remains the most common treatment for thymoma [13,14]; however, prognosis is highly dependent on tumor stage and histological subtype. The 5-year survival rate decreases from 93.3% in stage I disease to 43.4% in stage IV disease [12], while the 20-year survival rates of B2/B3 thymomas (59%/36%) are substantially lower than those of A/AB/B1 thymomas (100%/87%/91%) [15], indicating a particularly poor prognosis for higher-grade tumors. Owing to the lack of a clearly defined pathogenic mechanism, no effective preventive measures or universally applicable screening strategies for thymoma have been established to date. Nevertheless, the clinical diagnosis of thymoma remains challenging. On the one hand, its clinical manifestations are nonspecific, and no reliable molecular biomarkers are currently available for early screening, resulting in a substantial proportion of patients being diagnosed at an advanced stage [16]. On the other hand, current diagnosis relies primarily on imaging modalities such as computed tomography (CT); however, considerable overlap exists in the imaging characteristics of different anterior mediastinal lesions, making differential diagnosis difficult [17,18,19]. For lesions that remain difficult to differentiate, invasive procedures such as anterior mediastinal biopsy may be required, which not only increase patient morbidity but may also lead to misdiagnosis due to sampling bias [20]. Therefore, the identification of a molecular biomarker capable of enabling the early and accurate detection of thymoma represents an urgent unmet clinical need. A schematic overview of the major aspects of thymoma research, including etiology, prevention, malignant progression, diagnosis, treatment, and predictive modeling, as well as WHO histological subtypes (Type A, AB, B1, B2, and B3), is provided (Figure 1).

Multi-omics integration offers unique advantages for biomarker discovery in rare tumors. Conventional single-omics analyses are often constrained by limited sample sizes and consequently suffer from inadequate statistical power [21]. By jointly analyzing molecular data across multiple biological layers, including transcriptomics, proteomics, and epigenomics, multi-omics approaches enable cross-validation of candidate signals across distinct molecular levels and effectively reduce the false-positive rate associated with single-omics analyses. In this manner, biological depth can partially compensate for limitations imposed by small sample sizes [22,23,24]. Furthermore, the incorporation of computational approaches such as machine learning provides powerful analytical tools for efficiently identifying and validating key signals from these high-dimensional integrated datasets [25]. In the present study, we adopted such an integrative strategy and systematically analyzed multi-omics datasets from thymoma, followed by machine learning-based feature selection, ultimately identifying Plakophilin 1 (PKP1) as a candidate diagnostic biomarker.

PKP1 is a key component of the desmosomal plaque and a member of the armadillo repeat protein family, playing an important role in maintaining intercellular adhesion between epithelial cells [26,27,28]. Desmosomes are specialized intercellular junctional structures that connect the intermediate filament cytoskeletons of adjacent cells [29,30]. PKP1 contributes to the structural integrity of epithelial tissues by linking desmosomal cadherins to the intermediate filament network [31,32,33]. Notably, primary human thymic epithelial cells have been demonstrated to produce exosomes carrying tissue-restricted antigens [34]. Thymic epithelial cells express a broad repertoire of tissue-restricted self-antigens through mechanisms involving autoimmune regulator (AIRE) and other regulatory pathways. Exosomes derived from these cells may serve as potential vehicles for the stable release of such antigens into bodily fluids, thereby providing a biological basis for the detection of thymic epithelial cell-derived proteins in the peripheral circulation [35,36]. However, to date, no systematic studies have characterized PKP1 expression in thymoma tissues, examined its relationship with circulating PKP1 levels in peripheral blood, or comprehensively evaluated its clinical utility as a diagnostic or predictive biomarker for thymoma. In addition, aberrant PKP1 expression has been reported in several epithelial malignancies, and its overexpression has been significantly associated with overall survival, suggesting that PKP1 may play an important role in tumor initiation and progression [37].

In this study, we integrated multi-omics analyses with machine learning approaches to identify PKP1 as a potential diagnostic biomarker for thymoma with high diagnostic performance and tumor cell-enriched expression. These findings provide new insights into the development of highly sensitive and accurate diagnostic tools for the clinical detection of thymoma.

This schematic diagram illustrates six core research directions of thymoma: etiology, prevention, malignant progression, diagnosis, treatment, and prediction model construction. The bottom panel displays different thymoma subtypes (Type A, Type AB, Type B1, Type B2, and Type B3). The central inset depicts the anatomical location of the thymus within the thoracic cavity. Etiological factors include genetic predisposition, autoimmunity-related factors, environmental exposures, and other risk factors. Prevention strategies encompass risk factor avoidance, regular health screening, immunity enhancement, and smoking and alcohol cessation. The malignant progression panel shows the transition from stage I thymoma to invasive thymoma, while thymic carcinoma represents a distinct malignancy derived from thymic epithelium. Diagnostic approaches consist of imaging examinations, laboratory tests, and clinical evaluation. The main treatment modalities include surgery, radiotherapy, chemotherapy, and immunotherapy. Prediction strategies integrate imaging features, molecular markers, biomarkers, and multimodal artificial intelligence models.

## 2. Materials and Methods

### 2.1. Sources of Original Data

This study is a retrospective exploratory biomarker study aimed at characterizing the expression patterns and potential biological significance of PKP1 in thymoma. All bioinformatics data were obtained from public databases. To identify thymoma-related expression datasets, a systematic search was conducted in the GEO and TCGA databases using the keyword “thymoma.” The inclusion criteria were: (1) datasets containing gene expression profiles from both thymoma tissues and normal/adjacent normal thymic tissues; (2) samples with clear pathological diagnosis information; and (3) complete and accessible raw expression matrices. The exclusion criteria were: (1) insufficient sample size (fewer than 2 samples per group); (2) lack of definite pathological diagnosis of thymoma or missing diagnostic information; (3) severely missing data or incomplete expression matrices; and (4) in vitro cell line-based experimental datasets. Following these criteria, the transcriptomic datasets included GSE79978 (tumor samples, *n* = 13; normal samples, *n* = 3) and GSE177522 (tumor samples, *n* = 2; normal samples, *n* = 5) obtained from the Gene Expression Omnibus (GEO) database, as well as publicly available data from The Cancer Genome Atlas (TCGA) (tumor samples, *n* = 120; adjacent normal samples, *n* = 2). Among these datasets, GSE79978 and GSE177522 were used as the training set, while the TCGA dataset served as the validation set. To ensure the rigor of model evaluation, all batch correction, data normalization, feature screening, hyperparameter optimization, and model selection were performed exclusively within the training set; the TCGA dataset was used only for final model performance assessment and did not participate in any model construction procedures (see Section 2.4).

The DNA methylation dataset GSE218549 was retrieved from the GEO database (tumor samples, *n* = 113; normal samples, *n* = 9). Proteomic data (PXD039744) were obtained from the ProteomeXchange database (tumor samples, *n* = 15; adjacent normal samples, *n* = 11).

The single-cell RNA sequencing (scRNA-seq) dataset was obtained from https://singlecell.broadinstitute.org/single_cell/study/SCP1532 (accessed on 29 September 2025) and included four tumor samples from four patients with myasthenia gravis–associated thymoma, comprising a total of 33,725 cells (35,869,073 reads) [38].

Single-cell data analysis was performed using R version 4.4.2 via the Paratera (https://www.paratera.com/ accessed on 29 September 2025), while all other analyses were conducted using R version 4.5.0.

### 2.2. Transcriptome (Bulk RNA-Seq)

To obtain the gene expression matrix of the GSE177522 dataset, upstream transcriptomic analyses were performed. First, FastQC (v0.12.1) was used to assess the quality of the raw sequencing data. Subsequently, cutadapt (v4.4) was applied to remove adapter sequences and low-quality reads (with a base quality threshold of Q20), and reads with an excessive proportion of ambiguous bases (N bases) were filtered out, retaining clean reads of ≥50 bp for downstream analysis. The filtered reads were then aligned to the human reference genome (hg38) using HISAT2 (v2.2.1) [39]. Finally, FeatureCounts (v2.1.1) was used with the reference genome annotation files to count and generate the raw gene expression count matrix for each sample [40].

To further enhance the generalizability of the results, GSE177522 and GSE79978 were integrated for cross-dataset analysis, and batch effects were corrected using the ComBat-seq function from the sva package (v3.58.0) in R [41]. Subsequently, differentially expressed genes (DEGs) from both datasets that met the above criteria (|log_2_Fold Change| > 1, *p*.adjust < 0.05) were intersected. To ensure the accuracy of the intersection, all genes were matched using Gene Symbols as unified identifiers. These shared DEGs were identified using DESeq2 (v1.50.2), with thresholds set at |log_2_Fold Change| > 1 and *p*.adjust < 0.05 [42]. The results were visualized as volcano plots using ggplot2 (v3.5.2) (https://github.com/tidyverse/ggplot2 accessed on 15 August 2025). Heatmaps were generated using the heatmap function to visualize the expression patterns of these shared differentially expressed genes.

### 2.3. Proteomic and DNA Methylation Data Processing and Analysis

To validate the transcriptomic findings at the protein level, we retrieved the dataset PXD039744 from the ProteomeXchange public database, which contains mass spectrometry data from 15 thymoma tissues and their adjacent normal tissues. Based on the resulting protein expression matrix, differentially expressed proteins were identified using the limma R package (v3.64.1). Proteins with *p*.adjust < 0.05 and |log_2_Fold Change| > 1 were defined as significantly differentially expressed and were further classified into upregulated and downregulated groups.

To examine aberrant DNA methylation patterns in thymoma, DNA methylation data of 122 thymoma cases (normal: *n* = 9; tumor: *n* = 113) were downloaded from GEO (GSE218549). Quality control procedures were performed on the raw data: probes with missing values across all samples were removed; a 95% confidence ellipse was constructed based on the PC1 and PC2 coordinates of the 9 normal samples, and 56 tumor samples falling within this ellipse were excluded; a 95% confidence ellipse was then constructed for the remaining 57 tumor samples, and 3 tumor samples located outside the ellipse were excluded; subsequently, samples falling within the overlapping region of the two ellipses or deviating significantly from the main cluster in the principal component space were removed, resulting in 27 excluded samples (26 tumor and 1 normal). A total of 36 samples were ultimately included for analysis (normal: *n* = 8; tumor: *n* = 28). Differential methylation analysis between tumor and normal samples was performed using the R package ChAMP (v2.38.0) [43], with thresholds set at |Δβ| (difference in mean β values between tumor and normal groups) > 0.2 and *p*.adjust < 0.05, yielding lists of significantly hypermethylated and hypomethylated genes. Methylation probes located within the TSS1500 region, a promoter-proximal region upstream of the transcription start site (TSS), were specifically considered in the analysis. Genes showing concordant changes across the transcriptomic, proteomic, and DNA methylation levels were selected, including upregulated genes with hypomethylation and downregulated genes with hypermethylation. These genes were then subjected to machine learning to identify core candidates.

### 2.4. Machine Learning

To screen potential biomarkers from candidate genes, this study utilized the expression levels of 26 overlapping genes in the batch-corrected training set (GSE79978 and GSE177522, *n* = 23) as features, with sample type as the binary outcome variable to construct a predictive model for thymoma diagnosis. The 26 candidate genes were pre-identified through multi-omics intersection analysis before machine-learning model construction. To ensure the rigor of model development and evaluation and to prevent data leakage, a strict sequential workflow was implemented. First, batch effects between the two training datasets were corrected using ComBat-seq, followed by normalization. Multiple machine-learning algorithms were subsequently evaluated using the 26 candidate genes as input features. For each candidate algorithm, hyperparameters were optimized using five-fold cross-validation within the training set. The resulting candidate models were then evaluated in the TCGA set. The model achieving the highest AUC in the TCGA set was selected as the final model. Notably, because the TCGA set was used for model selection, its performance should not be considered fully independent evidence of model generalizability.

The final selected model, glmBoost + Elastic Net [alpha = 0.1], was subsequently subjected to an additional nested cross-validation analysis to assess its stability and robustness. A five-fold outer loop was used to evaluate model performance, while a five-fold inner loop was used for hyperparameter optimization. Within each fold, data standardization parameters were estimated using only the corresponding training subset and then applied to the held-out subset. Predictions from the outer test folds were aggregated to obtain the nested cross-validated AUC of the final model.

The nested cross-validation AUC was used as a supplementary, leakage-controlled estimate of the model’s internal performance, whereas the TCGA set was used for external model evaluation and model selection. Because TCGA performance contributed to the selection of the final model, further validation in an independent cohort is required to establish the generalizability of the model.

### 2.5. Single-Cell Transcriptomics (scRNA-Seq)

Single-cell RNA-sequencing data (SCP1532), comprising tumor tissues from four patients with MG-type thymoma, were obtained from the Single Cell Portal database. The 10× scRNA-seq data were processed using the Seurat package (v4.4.0) in R (https://github.com/satijalab/seurat accessed on 2 October 2025). Quality control was first performed by constructing a Seurat object with gene-level filtering parameters set as min.cells = 3 and min.features = 200. Subsequently, cell-level quality filtering was performed based on the percentile-based distribution of metrics within this dataset, rather than using universal fixed empirical cutoffs: the 5th and 95th percentiles of nFeature_RNA (574.0 and 4728.8, respectively) were used to remove cells with abnormally low or high transcriptomic complexity; the 95th percentile of nCount_RNA (3659.517) was applied to exclude potential doublets; and the 95th percentiles of percent.mt (2.0819036) and percent.rb (13.971344) were adopted to discard damaged, stressed, or low-quality cells. After quality filtering, a total of 33,725 cells were retained for downstream analyses. The data were then normalized using the NormalizeData function with the LogNormalize method, and the top 2000 highly variable genes were identified using the FindVariableFeatures function for principal component analysis (PCA). Unsupervised cell clustering was performed using the FindClusters function, and uniform manifold approximation and projection (UMAP) was applied via RunUMAP for nonlinear dimensionality reduction and visualization of clustering results. Cell cluster identities were annotated based on known canonical marker genes. A random seed was set to ensure reproducibility.

### 2.6. Computer Simulation of PKP1 Knockout

To investigate the functional role of PKP1 in thymoma, we performed virtual knockdown analysis on the tumor cell subpopulation within thymoma single-cell transcriptomic data using the R package scTenifoldKnk [44] (v1.0.3). By constructing a gene regulatory network (GRN) and simulating the knockout of the PKP1 node within this GRN, we implemented an unsupervised virtual knockdown approach with a random seed set to ensure reproducibility. This method effectively identifies genes that exhibit significant alterations in regulatory relationships as a result of PKP1 perturbation (with a threshold of adjusted *p* < 0.05), thereby allowing inference of its functional consequences.

### 2.7. Pathway Enrichment Analysis

GO enrichment analysis and KEGG pathway enrichment analysis were performed separately for upregulated and downregulated differentially expressed genes using the clusterProfiler package (v4.18.2) [45]. GO analysis simultaneously covered three ontologies: biological process (BP), cellular component (CC), and molecular function (MF). Significantly enriched biological processes and signaling pathways were identified with a threshold of *p*.adjust < 0.05, and representative GO terms and KEGG pathways were selected for visualization.

In this study, gene set enrichment analysis (GSEA) was conducted using the gseGO function in the clusterProfiler R package, with org.Hs.eg.db as the annotation database [46] and gene sets derived from the GO resource. The input for GSEA was a pre-ranked list of all genes sorted by log_2_Fold Change in descending order. After Benjamini–Hochberg (BH) correction, gene sets with FDR < 0.05 were considered significantly enriched. Immune-related significantly enriched terms were visualized using the enrichplot package (v1.30.4), with normalized enrichment score (NES), *p* values, and FDR values annotated.

### 2.8. Immunohistochemistry

Paraffin-embedded tissue samples from 25 thymoma samples and 8 adjacent normal samples (including one case of thymic adipose hyperplasia and one thymic cyst) were obtained from the First Affiliated Hospital of Henan University. The inclusion criteria were as follows: pathologically confirmed thymoma with complete clinicopathological data. The exclusion criteria were: preoperative radiotherapy, chemotherapy, or immunotherapy; concurrent other primary malignant tumors; and poor tissue quality that failed to meet the requirements for IHC detection.

Immunohistochemistry (IHC) was performed to assess the expression level and subcellular localization of Plakophilin-1 (PKP1). The specific steps are as follows: After routine dewaxing and rehydration of the tissue sections, they were placed in pH 9.0 ethylenediaminetetraacetic acid (EDTA) buffer and subjected to antigen retrieval via microwave heating. To block endogenous peroxidase activity, the sections were incubated with 3% hydrogen peroxide in methanol solution at room temperature. Following blocking with 3% bovine serum albumin, the sections were incubated overnight with the primary antibody against PKP1 (Proteintech, 22632-1-AP; dilution 1:200, Proteintech Group, Inc., Wuhan, China) in a humidified chamber at 4 °C. Subsequently, the sections were incubated with horseradish peroxidase (HRP)-conjugated goat anti-rabbit secondary antibody at room temperature for 50 min. The staining was developed using diaminobenzidine (DAB) substrate, and the cell nuclei were counterstained with hematoxylin.

Subcellular localization of PKP1 was evaluated by two independent observers who were blinded to the sample grouping. Sections were coded before evaluation, and the observers had no knowledge of the tumor or adjacent-normal status. Localization patterns were determined by the distribution of the DAB-positive (brown) signal; membranous staining was defined as brown signals along the cell membrane or cell borders (including dot-like or linear deposits at cell–cell contacts), whereas cytoplasmic staining was defined as diffuse or granular brown signals within the cytoplasm. Mixed and nuclear staining was recorded when present. Discrepancies were resolved by joint review.

Independent of the localization assessment described above, PKP1 expression levels were quantified objectively using ImageJ by an investigator blinded to the sample grouping. Images were acquired at 40× magnification from one systematically sampled tumor region per section. Image analysis was performed using ImageJ software (v1.54g; National Institutes of Health, USA). The color deconvolution plugin was applied to separate the DAB (brown) and hematoxylin (blue) channels. Positive staining (PKP1 immunoreactivity) was quantified by applying a fixed, predefined threshold to the DAB channel, which was kept consistent across all analyzed images. Results were expressed as the percentage of DAB-positive area relative to the total analyzed tumor area. Differences in PKP1-positive area percentages between thymoma and adjacent normal tissues were compared using a two-tailed Mann–Whitney U test, with statistical significance set at *p* < 0.05.

### 2.9. Statistical Analysis

All statistical analyses in this study were performed using R. Single-cell RNA-seq analysis was conducted in R (v4.4.2), while all other bioinformatic analyses and IHC quantitative statistics were performed in R (v4.5.0). The R packages and their corresponding versions used in each analytical step are specified in the respective method subsections.

Raw transcriptomic expression matrices were filtered to remove lowly expressed genes prior to downstream analysis. Continuous variables were presented as mean ± standard deviation (mean ± standard deviation). For IHC data, differences in PKP1-positive area percentages between thymoma and adjacent normal tissues were compared using a two-tailed Mann–Whitney U test. All statistical tests were two-sided, and *p* < 0.05 was considered statistically significant. Semi-quantitative IHC data exported from ImageJ (v1.54g) were imported into R for statistical analysis.

For bioinformatic differential expression analysis, DESeq2 (v1.50.2) was used for transcriptomic analysis with thresholds of |log_2_Fold Change| > 1 and adjusted *p*-value (*p*.adjust) < 0.05 after Benjamini–Hochberg correction. Differential proteomic analysis was performed using limma (v3.64.1) with identical cutoffs. Differential DNA methylation analysis was implemented via ChAMP (v2.38.0), with |Δβ| > 0.2 and *p*.adjust < 0.05 used to screen differentially methylated loci.

The area under the receiver operating characteristic curve (AUC) served as the primary metric to evaluate machine learning model performance. The 95% confidence intervals of AUC were estimated using the Bootstrap method with 1000 resamples.

The area under the receiver operating characteristic curve (AUC) served as the primary metric to evaluate machine learning model performance. The 95% confidence intervals of AUC were estimated using the Bootstrap method with 1000 resamples. Model training and hyperparameter optimization were performed via five-fold cross-validation within the training set. For gene set enrichment analysis (GSEA), FDR < 0.05 was defined as the threshold for statistical significance.

## 3. Results

### 3.1. Transcriptomic Analysis

After merging the GSE79978 dataset (13 thymic epithelial tumors and 3 normal samples) and the GSE177522 dataset (2 thymoma and 5 normal samples), differential analysis identified 1833 differentially expressed genes (DEGs) (|log_2_Fold Change| > 1, *p*.adjust < 0.05, Figure 2A), with 941 upregulated and 892 downregulated genes. The Pearson heatmap showed smaller variations within the normal group and greater variability within the tumor group (Figure 2B). The differential gene expression profile heatmap based on the top 50 genes clearly separated tumors from normal samples, indicating that transcriptional profiles could differentiate the two groups (Supplementary Figure S1A).

We performed Gene Ontology (GO) and Kyoto Encyclopedia of Genes and Genomes (KEGG) enrichment analyses on the DEGs. The KEGG and GO enrichment analysis of the upregulated DEGs (Figure 2C) revealed significant functional enrichment across the three major categories of GO: biological processes (BP), cellular components (CC), and molecular functions (MF). In BP, upregulated DEGs were notably enriched in cell adhesion-related processes, which may reflect active involvement of cell–cell adhesion and structural abnormalities in thymoma. CC analysis showed that upregulated DEGs primarily encode components of the extracellular matrix and various cell junction structures, consistent with significant remodeling of the extracellular microenvironment and intercellular junctions in thymoma. In MF, upregulated DEGs were mainly involved in extracellular matrix binding, cell adhesion mediator activity, and extracellular matrix structural constituents, revealing enhanced cell–cell and cell–matrix interactions in thymoma. KEGG pathway analysis revealed significant enrichment of upregulated DEGs in the integrin signaling pathway, immunoglobulin superfamily cell adhesion molecule (IgSF CAM) signaling pathway and cornified envelope formation pathway, pointing to possible links to tumor immune evasion and metastasis in thymoma.

The KEGG and GO enrichment analysis of the downregulated DEGs (Figure 2D) revealed significant functional enrichment. The downregulated pathways identified in the BP category are mainly related to core functions of the adaptive immune response, suggesting the presence of an immunosuppressive microenvironment in thymoma. Additionally, pathways involved in leukocyte cell–cell adhesion and regulation of leukocyte cell–cell adhesion were also significantly downregulated, reflecting the tumor’s potential mechanism of immune surveillance evasion. In the CC category, downregulated DEGs were significantly enriched in immunological synapse and external side of plasma membrane, which may reflect down-regulation of immune cell function. In the MF category, downregulated DEGs were primarily enriched in immune-related molecule binding functions, consistent with attenuated immune signaling from a protein interaction perspective. KEGG pathway analysis showed that the downregulated DEGs in thymoma were enriched in pathways such as the T cell receptor signaling pathway, and primary immunodeficiency, suggesting potential associations with immune dysfunction, immune cell interaction, and signaling abnormalities in thymoma.

Gene set enrichment analysis (GSEA) results indicated that immune-related pathways were significantly suppressed in thymoma, with genes related to T cell-mediated immunity and activation of immune response pathways being notably downregulated (Figure 2E,F). Other immune-related pathways also showed significant downregulation (Supplementary Figure S1B–E).

### 3.2. Proteomics and Epigenomic Analysis

First, principal component analysis (PCA) was performed on the PXD039744 dataset from the ProteomeXchange database, which included 11 adjacent normal samples and 15 tumor samples. The PCA clearly showed a distinct separation between thymoma and adjacent normal tissues, with minimal variation within the groups (Figure 3A). Differential analysis identified 1501 differentially expressed proteins (DEPs), with 1122 upregulated and 379 downregulated proteins (Figure 3B). The heatmap was generated by the top 100 DEPs, showing clear separation between tumor and adjacent normal tissues, indicating distinct protein expression profiles with minimal within-group variation (Figure 3C). To systematically explore the molecular mechanisms underlying thymoma development, GO and KEGG enrichment analysis were performed on the DEPs between the thymoma and adjacent normal groups.

GO enrichment analysis of upregulated DEPs indicates (Figure 3D) that upregulated proteins are significantly enriched in functional pathways related to intercellular connections, helicase activity, RNA splicing, and protein synthesis processing. In terms of BP, the core enrichment involves RNA splicing (GO:0008380), and protein-RNA complex assembly (GO:0022618). In CC category, the focus is primarily on spliceosomal complex (GO:0005681) and nuclear speck (GO:0016607). MF enrichment includes cadherin binding (GO:0045296), helicase activity (GO:0004386), and translation initiation factor activity (GO:0003743). KEGG pathway analysis (Figure 3E) reveals that upregulated DEPs are significantly enriched in cancer-related pathways and key signaling pathways, including the PD-L1 expression and PD-1 checkpoint pathway in cancer (hsa05235) and T cell receptor signaling pathway (hsa04660). Additionally, pathways involved in genetic information processing, such as DNA replication (hsa03030), spliceosome (hsa03040), as well as metabolic pathways like amino sugar and nucleotide sugar metabolism (hsa00520) and nucleotide metabolism (hsa01232), are also significantly enriched. These results suggest that upregulated DEPs are consistent with altered transcription and translation processes and activated cancer-related pathways, which may reflect epithelial tissue morphological abnormalities and pathological progression of thymoma.

GO enrichment analysis of downregulated DEPs indicates (Figure 3F) that downregulated proteins are significantly enriched in immune-related pathways, vesicle function, and protease activity-related pathways. In BP, core enrichment includes complement activation (GO:0006959) and humoral immune response (GO:0006959). In CC, downregulated DEPs are primarily enriched in secretory granule lumen (GO:0034774), vesicle lumen (GO:0031983), and extracellular matrix (GO:0031012). In MF, downregulated DEPs are significantly enriched in endopeptidase regulator activity (GO:0061135), and peptidase regulator activity (GO:0061134). KEGG pathway analysis (Figure 3G) reveals that downregulated DEPs are significantly enriched in immune-related pathways and cytoskeleton-related functions, including complement and coagulation cascades (hsa04610) and regulation of actin cytoskeleton (hsa04810). Additionally, metabolic pathways related to fatty acid metabolism (hsa01212), cholesterol metabolism (hsa04979), and citrate cycle (TCA cycle, hsa00020) are also involved. These results indicate that downregulated DEPs are enriched in functional categories related to immune responses, thymic microenvironment homeostasis, and metabolic processes, which may reflect an immunosuppressive microenvironment and possibly contribute to immune escape and pathological progression in thymoma.

Principal component analysis was also performed on the GSE218549 dataset, which included 8 normal and 28 thymoma samples, revealing clear separation between thymoma and normal tissues with minimal within-group variation (Figure 3H). Differential methylation analysis identified 7181 differentially methylated genes, with 1354 genes showing significant hypermethylation and 5827 genes showing significant hypomethylation (Figure 3I). A heatmap of the top 100 differentially methylated genes revealed distinct methylation profiles between tumor and normal tissues with minimal within-group variation and good consistency (Figure 3J). The intersection of upregulated genes from transcriptomic and proteomic data with downregulated methylated genes identified 25 genes (Supplementary Figure S1G), while the intersection of downregulated genes with hypermethylated genes identified 1 gene (Supplementary Figure S1H, Table S1).

### 3.3. Machine Learning for Diagnostic Model Development

To identify core genes, we selected intersecting genes from multiple omics datasets and constructed 56 machine learning models, evaluating their performance on the training set (*n* = 23) and an external The Cancer Genome Atlas (TCGA) validation set (*n* = 122) (Figure 4A). Based on the area under the ROC curve (AUC) values in the TCGA validation set, we determined that the glmBoost + Enet[alpha = 0.1] model was the most optimal (Table S2).

This model demonstrated strong predictive ability (training set AUC = 0.983, 95% confidence interval (CI): 0.944–1.000, sensitivity: 1.000 (95% CI: 0.796–1.000), specificity: 0.75 (95% CI: 0.409–0.929); validation set AUC = 0.983, 95% CI: 0.947–1.000, sensitivity: 1.000 (95% CI: 0.969–1.000), specificity: 0.500 (95% CI: 0.095–0.905)) (Figure 4B) and identified six key feature genes: IRF6, TRIP6, PKP1, CDH1, PBX1, and KRT1 (Table S3). 

In the training set, all six feature genes had AUC values greater than 0.7, with TRIP6, PKP1, CDH1, PBX1, and KRT1 achieving AUC values greater than or equal to 0.8, demonstrating excellent diagnostic performance (Figure 4C). In the external validation set, IRF6, TRIP6, PKP1, CDH1, and KRT1 maintained AUC values greater than 0.7, with TRIP6, PKP1, and KRT1 showing AUC values greater than 0.8, indicating strong generalization ability (Figure 4D).

Notably, PKP1 had a consistently high ROC value (training set AUC = 0.833, 95% CI 0.642–0.983; external validation set AUC = 0.942, 95% CI 0.896–0.979) and displayed significant expression differences (Figure 4E,F). TRIP6 also had a relatively high ROC value (training set AUC = 0.800, 95% CI 0.600–0.967; external validation set AUC = 0.900, 95% CI = 0.825–0.958), with significant expression differences (Supplementary Figure S2A,B). KRT1 showed consistently high ROC values (training set AUC = 0.983, 95% CI =0.925–1.000; external validation set AUC = 0.958, 95% CI = 0.883–1.000), but expression differences were not significant in the external validation set (Supplementary Figure S2C,D). IRF6, CDH1, and PBX1 had lower AUC values, and expression differences are shown in Supplementary Figure S2E–J.

To further evaluate the reliability and generalizability of our model, we performed nested cross-validation (CV) with a 5-fold outer loop and a 5-fold inner loop on the training set. The inner loop was used to optimize the regularization parameter (alpha) of the elastic-net model, while the outer loop was employed for unbiased performance estimation. The pooled AUC from the nested CV procedure was 0.900 (95% CI: 0.730–1.000), indicating favorable discriminative ability of the model within the training set (Supplementary Figure S2K). Furthermore, the independent external validation set (TCGA) was used to assess the generalization performance of the model, yielding an AUC of 0.983 (95% CI: 0.942–1.000) (Supplementary Figure S2L), which further corroborated the robust predictive capacity of our model.

### 3.4. Single-Cell Transcriptomics Analysis

To determine the cell types in which the feature genes selected by the machine learning model are predominantly expressed, we analyzed publicly available single-cell RNA sequencing data from tumor samples of 4 MG-type thymoma patients. After quality control, a total of 33,725 cells were retained for subsequent dimensionality reduction and clustering (resolution = 0.03). Using known cell markers, we identified 6 major cell types (Figure 5A,B), including: pre-B cells, Cytotoxic T cells, double-positive (DP) T cells, Macrophages, B cells, and Tumor cells.

The heatmap presents representative cluster-enriched marker genes that characterize the transcriptional identity of the distinct cell populations identified in the thymoma microenvironment. (Figure 5C). FOXN1 is a known biomarker that is specifically expressed in thymoma [47]. We then examined the distribution and expression of the 6 potential feature genes alongside FOXN1 across different cell types (Figure 5D–F).

The results revealed that TRIP6, KRT1, and CDH1 were expressed in tumor cells as well as other cell subtypes, lacking specificity. However, PBX1, IRF6, PKP1, and FOXN1 showed enriched expression in tumor cells (Supplementary Figure S3A–E).

Subsequently, we employed the scTenifoldKnk algorithm on thymoma single-cell data to construct a gene regulatory network (GRN) based on 415 tumor cells expression matrix. By removing the PKP1 node from this GRN to perform an in silico knockout simulation, we ultimately identified 85 genes exhibiting significant changes in regulatory relationships, filtered by adjusted *p* < 0.05. (Figure 5G, Table S4). Metascape enrichment analysis of these genes revealed significant over-representation of pathways associated with the ECM–integrin–cytoskeleton axis, including NABA ECM GLYCOPROTEINS (−log(adjusted *p*) = 21), Pid integrin1 pathway (−log(adjusted *p*) = 9.1), and Cytoskeleton in muscle cells (−log(adjusted *p*) = 8.9) among others (Figure 5H). These computational predictions suggest that genes within the predicted transcriptional network downstream of PKP1 are enriched for ECM–integrin–cytoskeleton-related signaling components.

### 3.5. Immunohistochemistry Results

To further confirm protein-level validation of differential PKP1 expressions, we performed immunohistochemistry (IHC) on 25 thymoma samples and 8 adjacent normal samples (including 1 case of thymic hyperplasia and 1 case of thymic cyst). The results showed that PKP1 was primarily localized in the cytoplasm of the cells. The positive expression of thymoma cells stained by the chromogenic reagent showed an obvious brown-yellow color, whereas positive staining in the nuclei and adjacent normal cells was relatively rare. The cell nuclei counterstained by hematoxylin showed a blue color.

PKP1 expression was significantly elevated in thymoma tissues (Figure 6A) but relatively low in adjacent normal thymic tissues (Figure 6B). Semi-quantitative analysis revealed a significant difference in PKP1 expression levels with statistical significance, consistent with the bioinformatics analysis results (Figure 6C, Table S5). Additionally, proteomic data showed that PKP1 protein abundance was significantly increased in thymoma (Figure 6D).

## 4. Discussion

Thymoma is a rare anterior-mediastinal malignancy derived from thymic epithelial cells. Although it ranks as the most common neoplasm in the anterior mediastinum, its low overall incidence limits large-scale clinical experience and impedes the development of reliable diagnostic and prognostic frameworks [48]. Thymomas display substantial biological heterogeneity with frequent indolent features: some are incidentally detected after years of clinical quiescence, while others manifest aggressive invasion, recurrence or distant metastasis [11]. Diagnostic difficulties are further aggravated by its anatomical site and non-specific manifestations. Early-stage lesions are often asymptomatic; when symptoms emerge, compressive signs including cough, dyspnea and superior vena cava syndrome overlap with other mediastinal diseases (lymphoma, germ-cell tumors, mediastinal cysts), resulting in clinical and radiological ambiguity [10,49]. Histologically, diverse subtypes and complex multicompartmental architecture hinder precise classification and grading [50]. Unlike many solid tumors, thymoma lacks well-validated specific serum biomarkers for non-invasive screening, diagnosis or surveillance, and tissue-derived molecular markers have not entered routine clinical application [51]. Furthermore, 20–30% of thymoma cases develop paraneoplastic autoimmune diseases (PNDs), predominantly myasthenia gravis [52]. Taken together, low incidence, biological heterogeneity, non-specific clinical features, histological complexity, shortage of serum biomarkers and concomitant immune dysregulation create substantial obstacles for thymoma prediction and diagnosis, highlighting the demand for robust molecular-driven diagnostic and prognostic models.

In this study, we employed a multi-omics approach, integrating transcriptomics, proteomics, methylation, and single-cell RNA sequencing to systematically reveal potential biomarkers for thymoma. We first conducted a differential analysis of two transcriptomic datasets from GEO, obtaining a list of genes potentially associated with thymoma and performing functional enrichment analysis on them. Next, we performed differential analyses at the protein and epigenetic levels to identify relevant genes. We then selected the common differential genes across the three omics layers. Subsequently, we constructed a machine learning diagnostic model using the intersected genes, identifying six potential biomarkers. These candidate genes were further examined for their expression patterns in single-cell RNA sequencing data. Finally, we chose PKP1, which holds greater research value, for IHC confirmation, verifying that PKP1 is significantly overexpressed in tumor tissues from thymoma patients, suggesting its potential as a diagnostic biomarker for thymoma. In summary, this study provides candidate genes for the precise diagnosis of thymoma.

In our machine learning diagnostic model, six feature genes were ultimately selected. Among them, IRF6, TRIP6, CDH1, PBX1, and KRT1 form a functional network by regulating epithelial cell adhesion, differentiation, cytoskeletal homeostasis, and transcriptional activation, with the core focus on epithelial tissue integrity and cell fate regulation [53,54,55,56,57,58].

Notably, PBX1, as a pioneer transcription factor, can bind to target gene sites in silent chromatin [59], laying the foundation for subsequent activation of genes related to cell differentiation. The transcriptional regulatory network it participates in, along with IRF6, influences epithelial cell fate determination. CDH1, a member of the epithelial cadherin family [60], is a key component of adherens junctions. By forming complexes with β-catenin, α-catenin, and others, CDH1 anchors the actin cytoskeleton and maintains tight cell adhesion and tissue polarity [61]. Its stable distribution on the cell membrane is essential for establishing epithelial barrier function. IRF6 regulates the membrane recycling and localization of E-cadherin, thus maintaining the stability of adherens junctions between cells [62]. Meanwhile, KRT1, as a marker of terminal epithelial differentiation [63], depends on normal differentiation pathways. TRIP6 interacts with proteins such as MAGI-1b and PTEN phosphatases to compete for β-catenin binding sites, indirectly regulating CDH1-mediated cell adhesion [64]. It also participates in actin cytoskeleton rearrangement, influencing cell migration and invasion, complementing IRF6’s role in adhesion regulation [65].

This functional network is ultimately integrated through PKP1 (plakophilin 1). PKP1, a member of the armadillo repeat family of proteins [66], is a key structural component of desmosomal plaques.PKP1 interacts with desmosomal core proteins like desmoglein 1 or desmocollin 1, and desmoplakin, via its Armadillo repeat domain, to stabilize desmosomal structures and recruit cytoskeletal linkers [28], which are crucial for maintaining strong mechanical adhesion between keratinocytes [67]. On one hand, PKP1 synergizes with CDH1-mediated adherens junctions to stabilize epithelial cell adhesion, complementing IRF6 and TRIP6 in adhesion regulation [68]. On the other hand, PKP1 can also stabilize the desmosomal plaque structure and specifically regulate the clustered aggregation of Dsg3 (desmoglein 3) [69]. Together with the intermediate filament network formed by KRT1, it strengthens the mechanical integrity of epithelial tissue and resists mechanical stress. Additionally, PKP1 has non-adhesion-dependent translational regulatory functions. It can enhance protein synthesis by binding to eIF4A1 and promoting the assembly of the eIF4F complex [70], thus promoting abnormal cell proliferation. The signaling pathways involved likely intersect with the transcriptional regulation of PBX1 and the anti-apoptotic signaling of TRIP6, collectively maintaining epithelial cell homeostasis. Therefore, PKP1 becomes a pivotal hub connecting cell adhesion, structural support, and signal transduction. In this study, through the differential gene enrichment analysis of transcriptomic data, we found that cell adhesion-related pathways, including PKP1, were significantly upregulated in thymoma. Moreover, in the single-cell sequencing data, PKP1 was specifically enriched in the tumor cluster, further supporting its potential role as a biomarker for thymoma.

Unlike the current focus of machine learning that is primarily on radiomics data [71,72], this study innovatively applies 56 different machine learning models to identify core genes.

Machine learning has the advantages of efficiently processing high-dimensional data, accurately mining potential associations, strong reproducibility, and effectively reducing human errors [25]. Compared with traditional analysis methods [73], machine learning enables more precise feature selection from complex omics data. We identified the best-performing diagnostic model combination—the glmBoost + Enet[alpha = 0.1] model, which achieved an AUC of 0.983. Based on this optimal model, we identified a set of feature genes closely related to thymoma, thus enhancing the accuracy and reliability of the results and providing stronger support for thymoma diagnosis. Additionally, we integrated multi-omics data and prioritized candidate genes showing concordant alterations across the transcriptomic, proteomic, and epigenetic layers, reducing bias from single-omics analysis and confirming the reliability and specificity of the research results. This approach provided complementary evidence across multiple omics layers by identifying concordant alterations in PKP1 at the transcriptomic, proteomic, and DNA methylation levels, offering a more integrated view of the molecular landscape of thymoma. Importantly, these concordant patterns were interpreted as cross-omics associations supporting candidate-gene prioritization rather than direct evidence of epigenetic regulation. Moreover, single-cell omics clearly elucidated the molecular characteristics and cellular differences in various cell clusters, identified the cellular localization and expression abundance of PKP1, and reduced the masking effects caused by bulk-tissue averaging. By identifying the cell populations in which PKP1 was preferentially enriched, single-cell analysis provided additional cell-level evidence supporting biomarker validation. Together, these complementary analyses established a framework in which multi-omics data prioritized candidate biomarkers and single-cell analysis further characterized their cellular distribution, enhancing the biological interpretation and robustness of PKP1 as a candidate biomarker.

In summary, our biomarker combines mRNA, methylation, proteomics, and single-cell sequencing data, offering a relatively complete pathological landscape of thymoma, minimizing errors caused by single omics factors or other biological influences, and promoting more accurate thymoma identification. The integration of these multi-layered pieces of information enhances the overall characterization of the thymoma disease state, making the diagnosis more precise.

However, this study does have some limitations:

First, the data are sourced from public databases, including two GEO mRNA datasets, one methylation dataset, TCGA RNA data, ProteomeXchange proteomics data, and Single Cell database single-cell data. Notably, the TCGA validation set included 120 tumor samples but only 2 normal samples, and this pronounced imbalance may compromise the stability of the specificity estimate. The sensitivity was 1.000 (95% CI: 0.969–1.000), while the specificity was 0.500 (95% CI: 0.095–0.905). Despite this limitation, we regard the TCGA dataset as a valuable external validation of our findings. Future studies incorporating more balanced sample compositions will be essential to further consolidate the clinical evidence.

Second, the IHC control group comprised adjacent non-neoplastic thymic tissue but nevertheless included one case of thymic adipose hyperplasia and one case of thymic cyst. Although peritumoral tissue is a widely accepted surrogate in clinical practice, it may not fully recapitulate the normal thymic milieu, since the tumor microenvironment can elicit reactive changes in the surrounding parenchyma. The limited number of control samples restricted the statistical power and generalizability of the differential comparisons.

Third, regarding the IHC validation, only one systematically sampled tumor region per section was analyzed. This sampling strategy was adopted to ensure objective and consistent quantification, since all images were captured using a fixed, predefined threshold; nevertheless, given the potential intratumoral heterogeneity of PKP1 expression, a single region may not fully represent the whole tumor, and multi-region or whole-slide analyses in larger cohorts will be needed to further verify the robustness of our findings.

Fourth, all four single-cell samples were derived from MG-associated thymoma, which may limit the generalizability of our findings to other thymoma subtypes or non-MG thymomas. Future studies incorporating normal thymic tissues and a broader spectrum of thymic epithelial tumor subtypes will be required to further validate the specificity and clinical utility of the identified biomarkers.

Fifth, the six candidate genes, including PKP1, were identified through comparisons between thymoma and normal or adjacent non-tumorous tissues, and their enriched expression in tumor cells was further validated by single-cell transcriptomic analysis. Therefore, PKP1 should currently be regarded as a potential tissue-level biomarker for thymoma rather than a definitive tool for differential diagnosis. Further validation in larger, independent, and clinically well-characterized cohorts, including other anterior mediastinal tumors and lesions, is warranted to further assess its diagnostic performance.

Finally, although we have identified PKP1 as a potential biomarker for thymoma diagnosis, its precise mechanism of action in thymoma remains to be further investigated.

While our IHC data demonstrate elevated PKP1 protein abundance in thymoma tissue, several barriers remain for translating PKP1 into a circulating serum-based biomarker. First, the serum stability and effective half-life of circulating PKP1 have not been characterized. Even robust tissue overexpression does not guarantee measurable, stable protein levels in peripheral blood, as proteolytic degradation in serum, variable tumor release kinetics, and renal clearance may substantially alter circulating concentrations [74,75]. Future stability experiments using recombinant PKP1 in human serum under different temperature and freeze–thaw conditions will be required to evaluate its pre-analytical robustness and estimate its in vitro half-life before serum-based clinical utility can be justified.

Future research will focus on the following directions: First, constructing a PKP1 gene knockout model and using in vivo experiments to clarify the specific role of PKP1 in thymoma tumorigenesis and progression, as well as elucidating its involvement in the molecular mechanisms of thymoma development. Second, expanding clinical research cohorts and integrating multi-center clinical sample data to systematically validate the correlation between PKP1 expression levels and thymoma patient recurrence, metastasis, prognosis, and response to targeted therapy, further confirming its clinical value as a biomarker for thymoma diagnosis, prognosis assessment, and therapeutic targeting. Lastly, promoting the clinical translation of PKP1-related research findings by integrating metabolomics and radiomics to construct a multi-modal diagnostic system, which will further enhance the precision diagnosis and treatment of thymoma. Metabolomics can capture dynamic changes in metabolites during thymoma tumorigenesis and progression, revealing the association between PKP1 and thymoma-related metabolic reprogramming. Through radiomics, we can explore the inherent link between thymoma imaging features and molecular markers, addressing the limitations of invasive clinical detection.

## 5. Conclusions

This study integrated multi-omics with machine learning, single-cell sequencing, virtual knockout, and IHC to identify a diagnostic biomarker for thymoma. Among the six core genes, PKP1 showed robust diagnostic performance in both training and validation sets, with tumor-enriched expression, potential ECM–integrin–cytoskeleton axis regulation, and protein overexpression confirmed by single-cell, in silico, and IHC analyses. These multi-omics patterns represent associations supporting candidate gene prioritization rather than direct evidence of epigenetic regulatory mechanisms. Overall, PKP1 is a reliable tissue-level biomarker for early thymoma diagnosis, although clinical validation in larger cohorts is warranted.

## Figures and Tables

**Figure 1:**
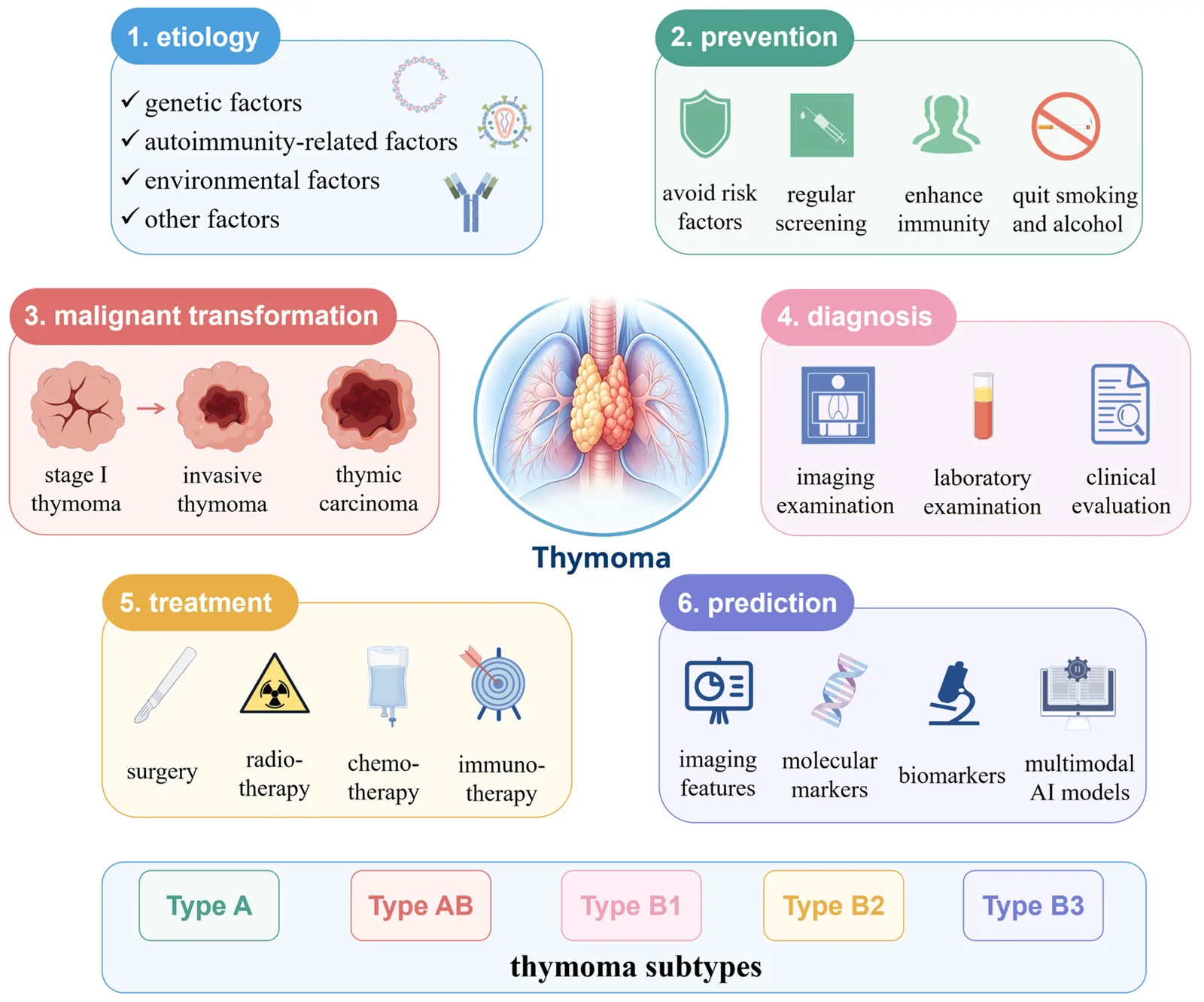
Overview of key components in thymoma research.

**Figure 2:**
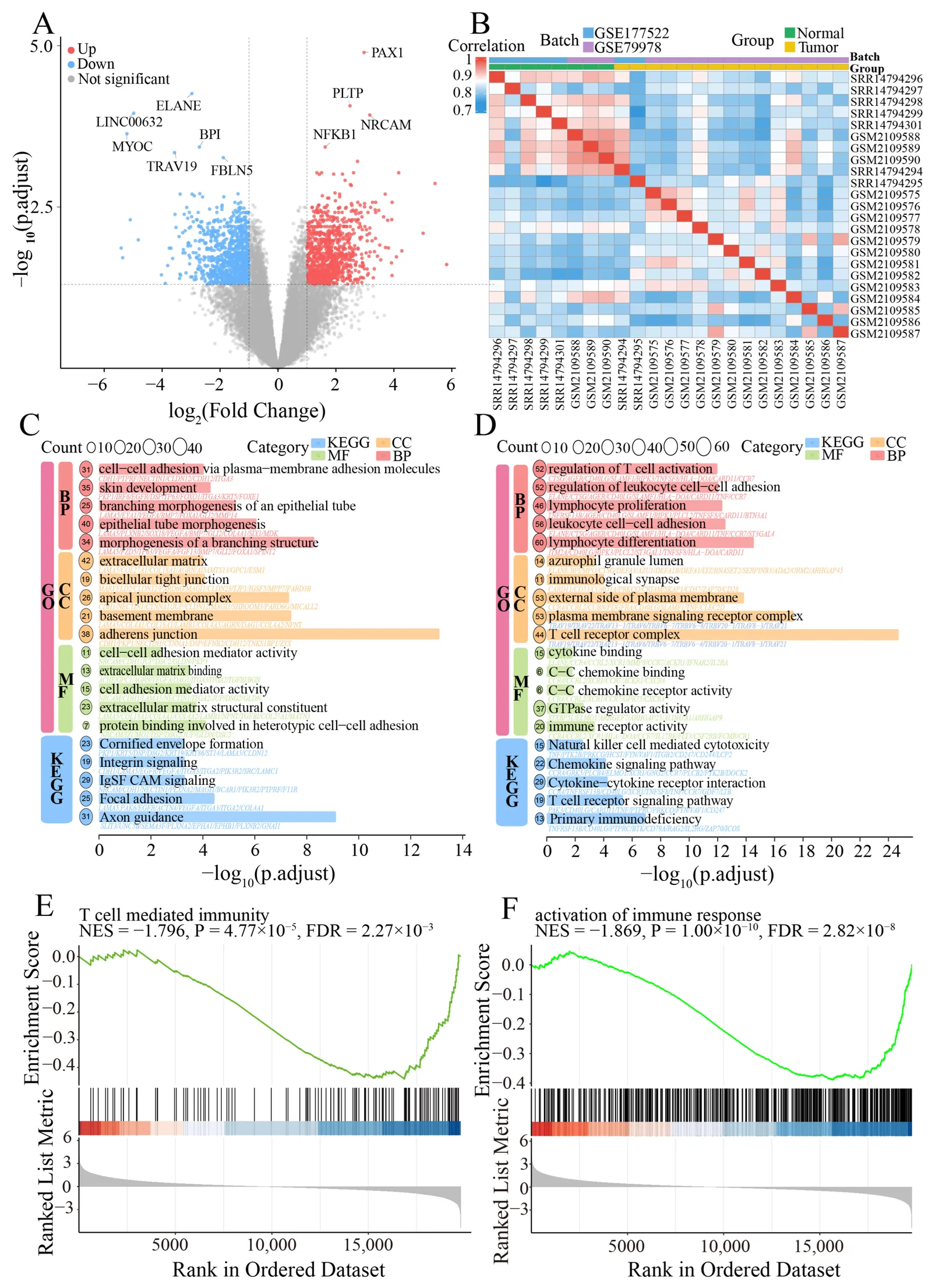
Integrated transcriptomic dataset screening and functional feature analysis of thymoma differential genes. (**A**): Volcano plot of DEGs between thymoma and normal tissues in the merged GSE79978 (*n* = 13 thymoma vs. 3 normal) and GSE177522 datasets (*n* = 2 thymoma vs. 5 normal) (selection criteria: |log_2_Fold Change| > 1, *p*.adjust < 0.05). Red points represent significantly upregulated genes (941), blue points represent significantly downregulated genes (892), and grey points represent genes without significant differences. (**B**): Pearson correlation heatmap between samples, reflecting the correlation between samples (red indicates high correlation, blue indicates low correlation). (**C**): KEGG and GO enrichment analysis of upregulated DEGs from the merged dataset. The functional enrichment results across the three main categories in GO, including BP, CC, and MF, are presented. The numbers on the left indicate the number of DEGs included in each enrichment entry. (**D**): KEGG and GO enrichment analysis of downregulated DEGs from the merged dataset. The functional enrichment results across the three main categories in GO, including BP, CC, and MF, are shown. The numbers on the left indicate the number of DEGs included in each enrichment entry. (**E**): GSEA results for the T cell-mediated immunity pathway (normalized enrichment score (NES) = −1.796, *p* = 4.77 × 10^−5^ false discovery rate (FDR) = 2.27 × 10^−3^), showing overall downregulation of this pathway, indicating immune-related functional suppression in thymoma. (**F**): GSEA results for the activation of immune response pathway (NES = −1.869, *p* = 1.00 × 10^−10^, FDR = 2.82 × 10^−8^), showing significant downregulation of this pathway, indicating immune-related functional suppression in thymoma.

**Figure 3:**
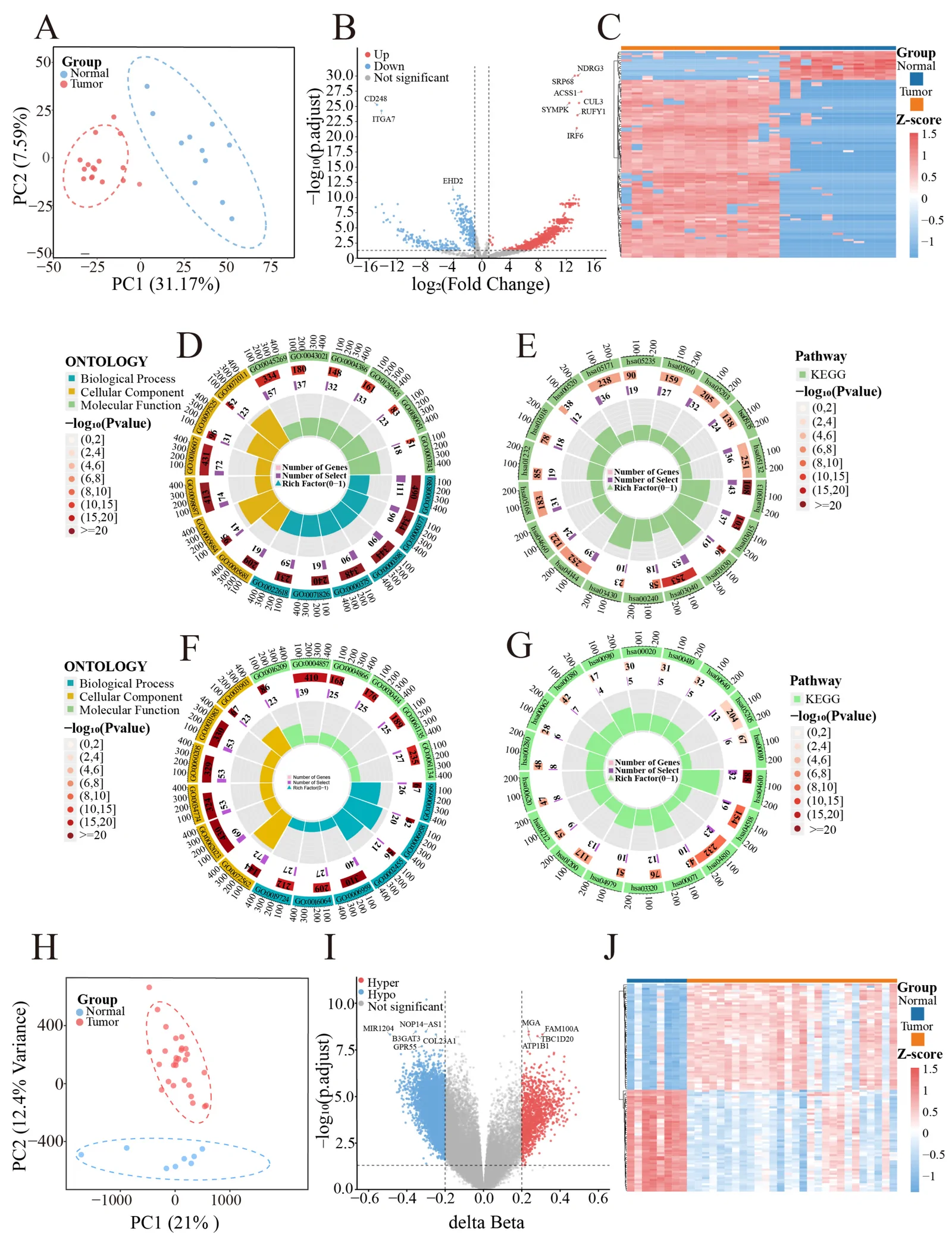
Proteomics and Epigenomics Analysis. (**A**): The PCA scatter plot of the PXD039744 dataset, where each point represents a sample. Blue points indicate adjacent normal samples (*n* = 11), while red points represent thymoma samples (*n* = 15). (**B**): Volcano plot depicting differentially expressed proteins between adjacent normal tissues and thymoma tissues(|log_2_Fold Change| > 1, *p*.adjust < 0.05). Red points on the right represent upregulated proteins (1122), and blue points on the left represent downregulated proteins (379). The x-axis corresponds to log_2_Fold Change, and the y-axis represents −log_10_(*p*.adjust). (**C**): Heatmap illustrating the expression patterns of the top 100 differentially expressed proteins (|log_2_Fold Change|) in adjacent normal tissues and thymoma tissues. Red indicates upregulated expression, and blue indicates downregulated expression. (**D**): A circular plot of GO functional enrichment analysis for upregulated proteins based on proteomics data. Six pathways were selected from each of the three major categories of GO enrichment: BP, CC, and MF. (**E**): A circular plot of KEGG functional enrichment analysis for upregulated proteins based on proteomics data. (**F**): A circular plot of GO functional enrichment analysis for downregulated proteins based on proteomics data. Six pathways were selected from each of the three major categories of GO enrichment: BP, CC, and MF. (**G**): A circular plot of KEGG functional enrichment analysis for downregulated proteins based on proteomics data. (**H**): PCA scatter plot of the GSE218549 dataset. Each point represents a sample, with blue indicating normal tissues (*n* = 8) and red indicating thymoma tissues (*n* = 28). (**I**): Volcano plot displaying differentially methylated probes, with red showing significantly hypermethylated probes and blue showing hypomethylated probes (|deltaBeta| > 0.2, *p*.adjust < 0.05). (**J**): Heatmap of the top 100 differentially methylated genes based on |deltaBeta|. Rows represent individual genes, and columns represent individual samples. The color of each small square indicates the methylation level, with red indicating high methylation and blue indicating low methylation.

**Figure 4:**
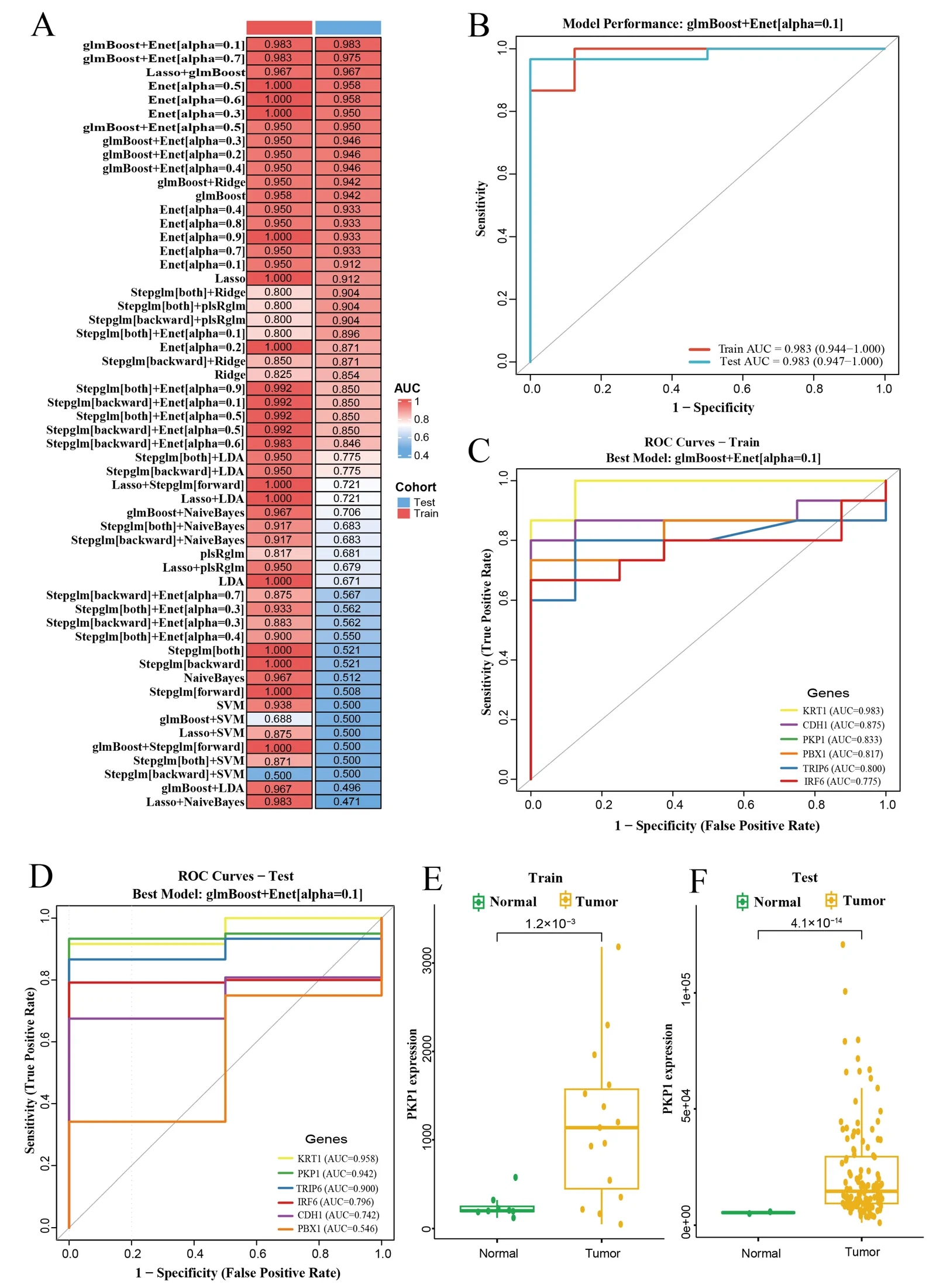
Machine Learning Model Selection and Validation of the Core Biomarker PKP1. (**A**): Heatmap of performance for multiple machine learning models, showing the AUC values for different algorithms in both the training set (red columns, *n* = 15 thymoma vs. 8 normal) and TCGA validation set (blue columns, *n* = 120 thymoma vs. 2 adjacent normal). The gradient color scale reflects the AUC value (red represents AUC = 1, blue represents AUC = 0.4). (**B**): Receiver operating characteristic (ROC) curves for the best-performing model (glmBoost + Enet[alpha = 0.1]) in both the training set and the external validation set AUC for the training set is 0.983 (95% CI: 0.944–1), and AUC for the external validation set is also 0.983 (95% CI: 0.947–1). (**C**): Diagnostic performance of IRF6, TRIP6, PKP1, CDH1, PBX1, and KRT1 in the training set, as assessed by ROC curves. (**D**): Diagnostic performance of IRF6, TRIP6, PKP1, CDH1, PBX1, and KRT1 in the external validation set, as assessed by ROC curves. (**E**): Boxplot showing the expression difference in PKP1 in the training set (*n* = 15 thymoma vs. 8 normal), where PKP1 expression is significantly higher in tumor tissues compared to normal tissues (*p* = 1.2 × 10^−3^, *t*-test). (**F**): Boxplot showing the expression difference in PKP1 in the external validation set (*n* = 120 thymoma vs. 2 adjacent normal), confirming the significantly higher expression in tumor tissues compared to normal tissues (*p* = 4.1 × 10^−14^, *t*-test).

**Figure 5:**
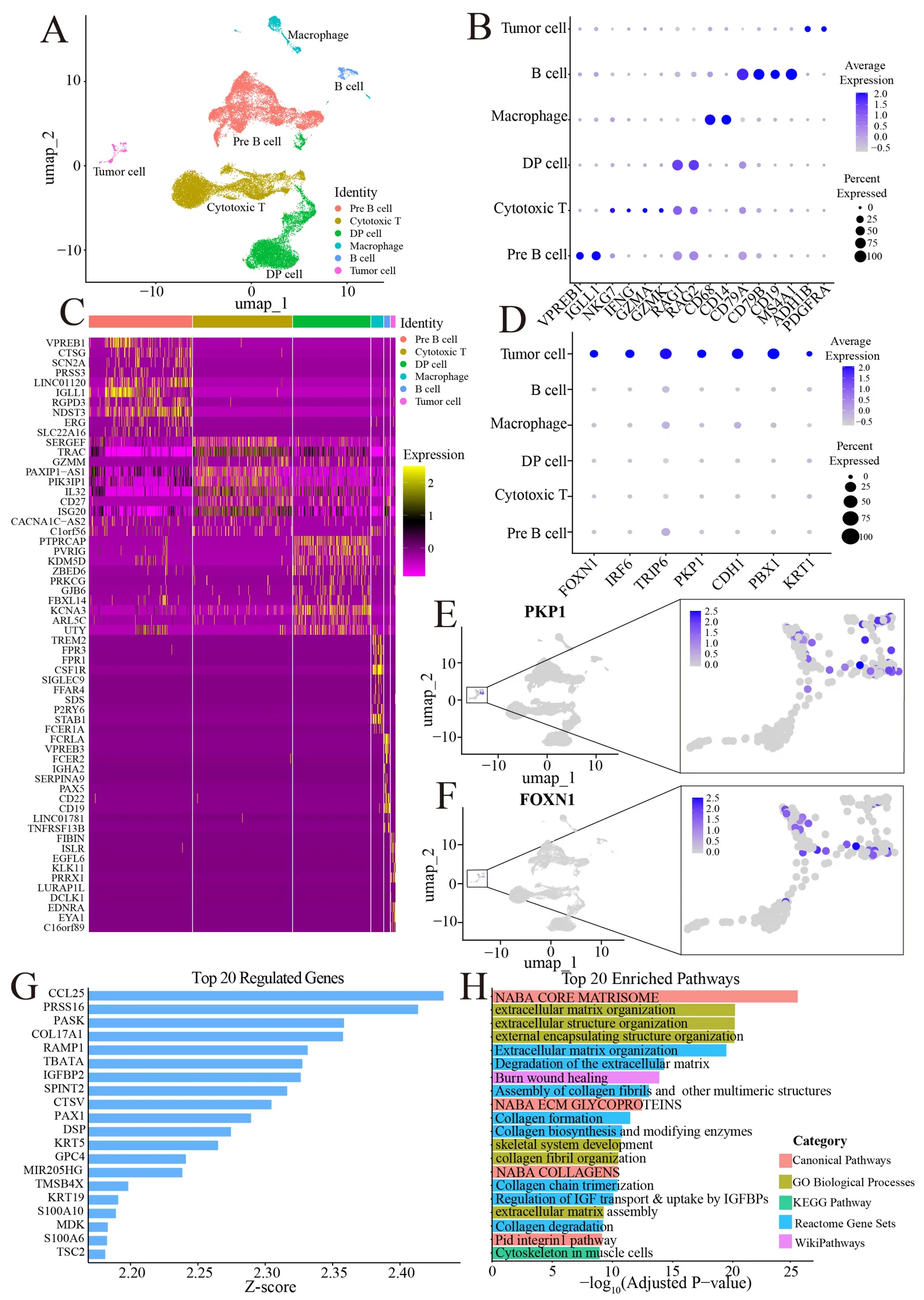
Single-Cell Data Analysis and Distribution of Core Genes Across Cell Clusters. (**A**): UMAP (Uniform Manifold Approximation and Projection) plot visualizing the highly structured cellular composition within the tumor microenvironment. Each point represents a single cell, and the color corresponds to the annotated cell type shown in the legend. A total of 33,725 cells from four patients with MG-type thymoma were analyzed, revealing six major cell populations: pre-B cells, cytotoxic T cells, DP T cells, macrophages, B cells, and tumor cells. (**B**): Dotplot showing the expression of marker genes across the six major cell types. Color scale: average expression levels; dot size: proportion of expressing cells. (**C**): Heatmap of the top 10 most significantly differentially expressed marker genes in each cell cluster, ranked by log_2_Fold Change. Rows represent genes, and columns represent cell clusters, showing gene-specific expression across the different cell types. (**D**): Dotplot reveals the average expression levels of IRF6, TRIP6, PKP1, CDH1, PBX1, and KRT1 across different clusters. (**E**): Feature plot shows the localization and expression levels of PKP1 across different clusters. PKP1 is enriched in the tumor cluster., with almost no expression in other clusters. (**F**): Feature plot shows the localization and expression levels of FOXN1 across different clusters. FOXN1 is highly enriched in the tumor cluster., with almost no expression in other clusters. (**G**): Bar plot shows the top 20 perturbation-responsive genes identified from PKP1 virtual knockdown were ranked by Z-score. A larger absolute Z-score indicates a more pronounced effect of PKP1 loss on the gene’s regulatory relationships. (**H**): Functional enrichment analysis was performed using Metascape on the 85 perturbation-responsive genes identified from the PKP1 virtual knockdown. Representative significantly enriched pathways and terms (adjusted *p* < 0.05) are displayed.

**Figure 6:**
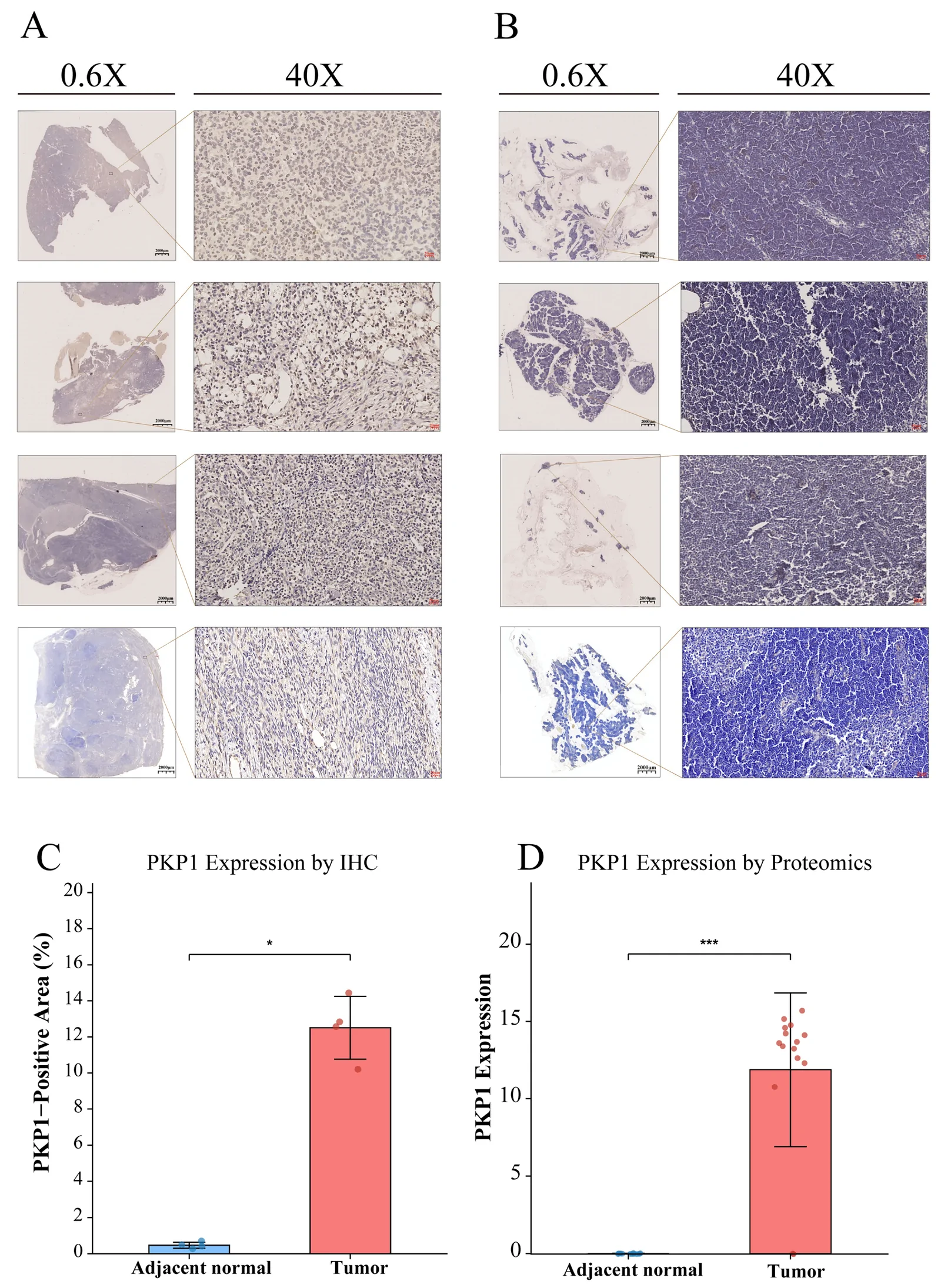
Thymoma Immunohistochemistry and Expression Differences in PKP1. (**A**): Immunohistochemical staining of thymoma tissue at low magnification (0.6×) and high magnification (40×). PKP1 is primarily localized in the cytoplasm, with positive staining areas appearing brown-yellow. (**B**): Immunohistochemical staining of adjacent normal tissue. PKP1 expression is low, with significantly reduced cytoplasmic positive signal, and the main color is blue from hematoxylin counterstaining. (**C**): Semi-quantitative analysis bar chart showing that the percentage of PKP1-positive areas in tumor tissue is significantly higher than in adjacent normal tissue (*n* = 4 thymoma vs. 4 adjacent normal, * *p* < 0.05, Mann–Whitney U test). (**D**): Proteomics data showing the PKP1 expression boxplot, confirming significantly higher expression of PKP1 in tumor tissues (*n* = 15 thymoma vs. 11 adjacent normal, *** *p* < 0.001, Mann–Whitney U test).

## Data Availability

The transcriptomic and methylation data utilized in this study were sourced from the Gene Expression Omnibus (GEO, https://www.ncbi.nlm.nih.gov/geo/ accessed on 13 August 2025) and The Cancer Genome Atlas (TCGA, https://www.cancer.gov/ccg/research/genome-sequencing/tcga accessed on 17 August 2025) databases. Proteomic data were obtained from ProteomeXchange (https://www.proteomexchange.org/ accessed on 20 August 2025), and single cell data were retrieved from the Single Cell Portal database (https://singlecell.broadinstitute.org/single_cell accessed on 29 September 2025). The analysis code supporting the findings of this study is publicly available at https://github.com/we8879/Thymoma-Multi-Omics-Biomarker-Discovery (accessed on 5 September 2026).

## Supplementary Materials

The following supporting information can be downloaded at: https://www.mdpi.com/article/10.3390/biomedicines14102151/s1, Figure S1: Differential Gene Identification, GSEA Enrichment Analysis, and Multi-Omics Intersection Gene Integration; Figure S2: Differential Expression Validation of Core Genes in Training and Validation sets; Figure S3: Expression Distribution of Feature Genes in Single-Cell Subpopulations; Table S1: List of the 26 candidate genes identified through multi-omics integration; Table S2: AUC values of the 56 machine learning models on the training set and the TCGA validation set; Table S3: Performance of the six key feature genes in the training set and the TCGA validation set; Table S4: Genes associated with PKP1 identified by in silico knockout; Table S5: Semi-quantitative analysis of PKP1 expression in thymoma and adjacent normal thymic tissues.
