# Supplementary material for: Integrative Multi-Omics and Machine Learning Unveil Plakophilin 1 as a Robust Diagnostic Biomarker for Thymoma
Source: Biomedicines. 2026 Sep 23;14(10):2151. doi: 10.3390/biomedicines14102151 (PMC13701948; doi:10.3390/biomedicines14102151)
Supplement: Supplementary file 1 [file biomedicines-14-02151-s001.zip › Supplementary Materials.pdf]

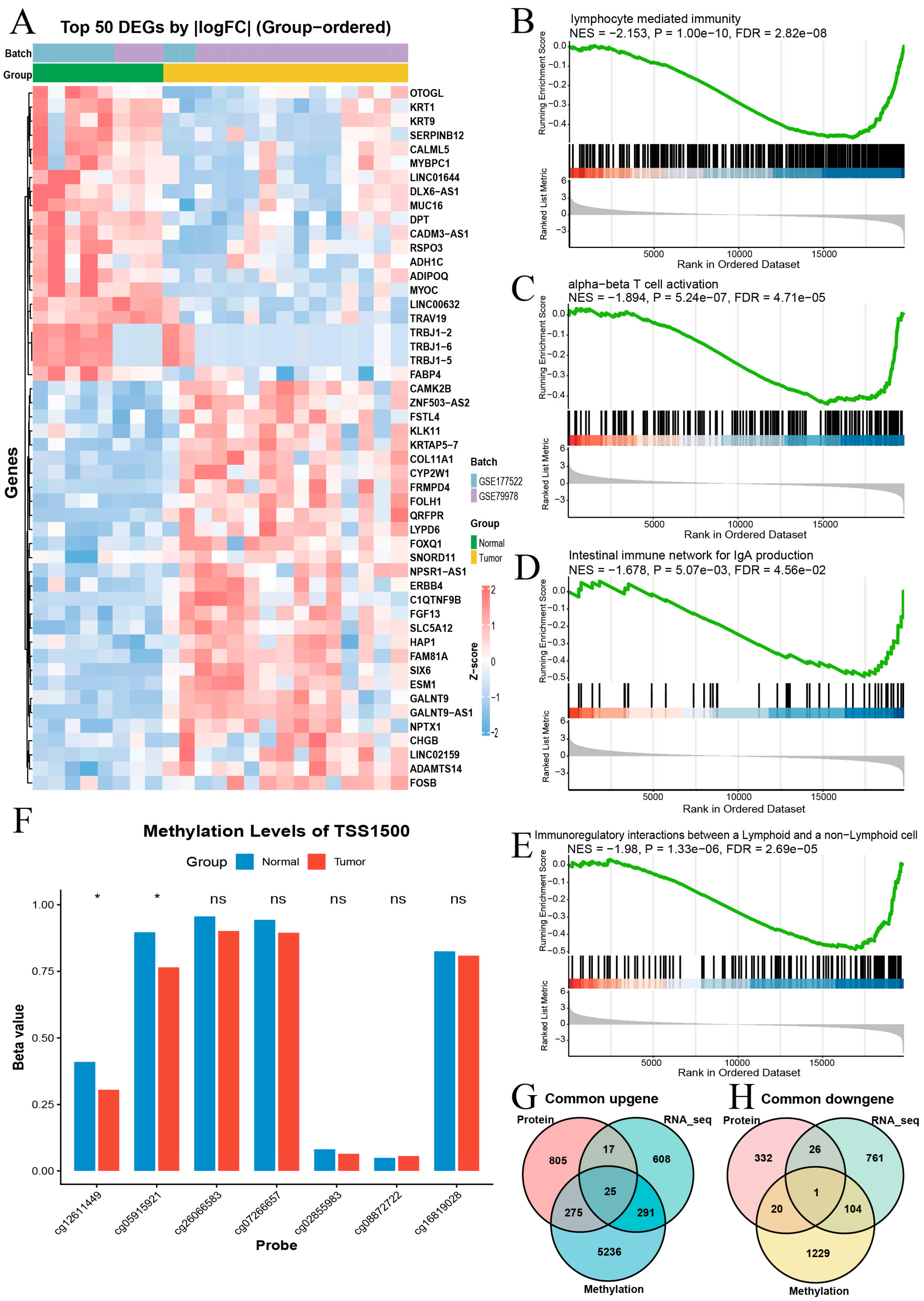

Figure S1: Differential Gene Identification, GSEA Enrichment Analysis, and Multi-Omics Intersection Gene Integration. (**A**): Heatmap of the expression of the top 50 differential genes based on |log_2Fold Change|. Red indicates upregulated gene expression, while blue indicates downregulated gene expression. (**B**): GSEA results show that the αβ T cell activation pathway has NES = -1.894, $p = 5.24 \times 10^{-7}$, FDR = 4.71×10^{-5}. (**C**): GSEA results show that the lymphocyte mediated immunity pathway has NES = -2.153, $p = 1.00 \times$ 10−10, FDR = 2.82×10^{-8}. (**D**): GSEA results show that the Intestinal immune

network for IgA production pathway has NES = -1.678, $p = 5.07 \times 10^{-3}$, FDR = 4.56×10^{-2}. (**E**): GSEA results show that the Immunoregulatory interactions between a Lymphoid and a non-Lymphoid cell pathway have NES = -1.98, $p = 1.33 \times 10^{-6}$, FDR = 2.69×10^{-5}. (**F**):Methylation levels of PKP1-associated probes within the TSS1500 region. The β values of seven methylation probes annotated to PKP1 within the TSS1500 region are shown for normal and thymoma tissues. Statistical comparisons were performed using the Wilcoxon rank-sum test (* $p < 0.05$; ns, not significant). (**G**): A Venn diagram is generated by intersecting upregulated genes from transcriptomics and proteomics with downregulated genes from methylation data, resulting in 25 common genes. **(H)**: A Venn diagram is generated by intersecting downregulated genes from transcriptomics and proteomics with upregulated genes from methylation data, resulting in 1 common gene.

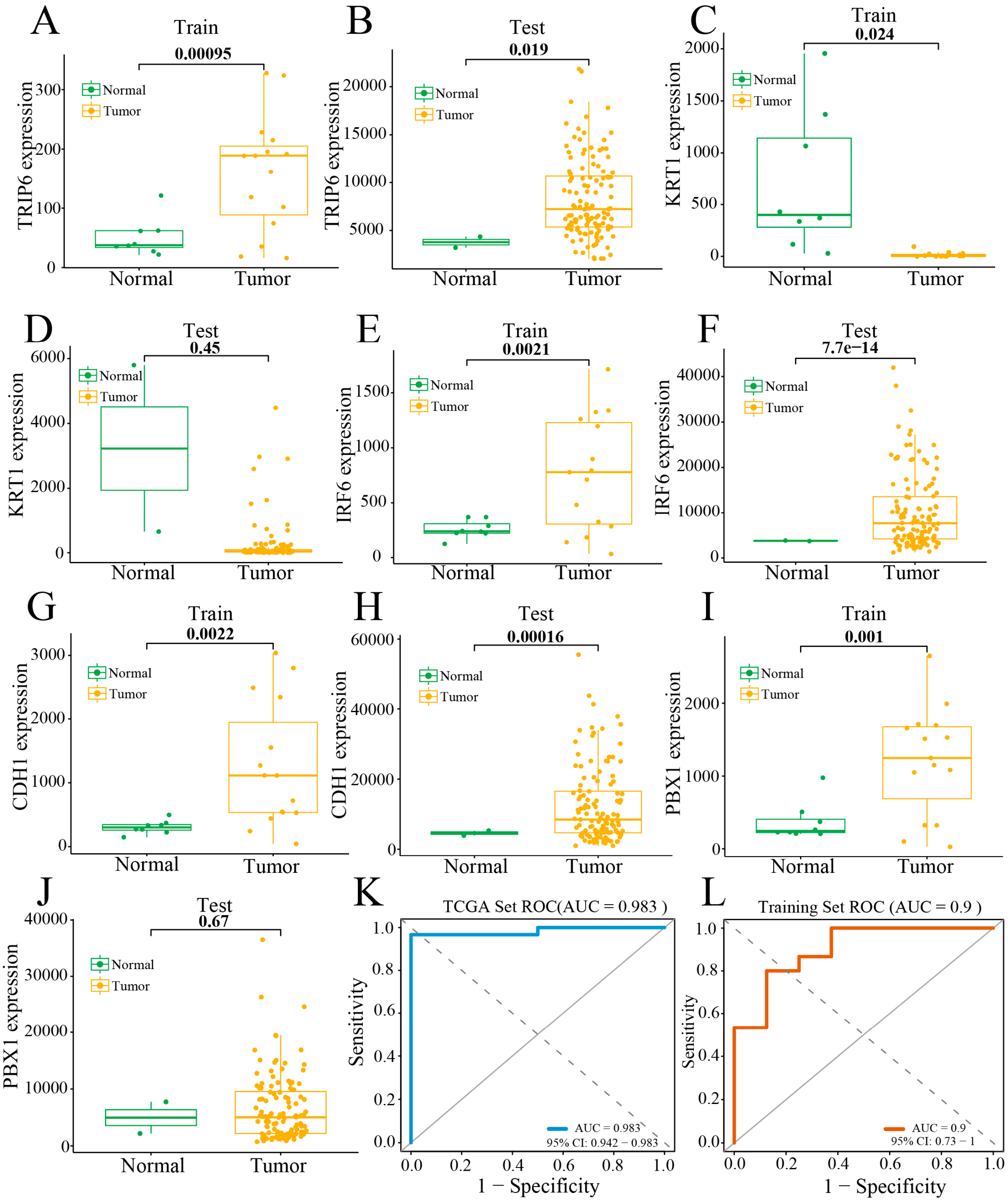

Figure S2: Differential Expression Validation of Core Genes in Training and Validation sets (**A**): Boxplot of TRIP6 expression differences in the training set, $p = 9.5 \times 10^{-4}$; t-test. (**B**): Boxplot of TRIP6 expression differences in the TCGA set, $p = 1.9 \times 10^{-2}$; t-test.. (**C**): Boxplot of KRT1 expression differences in the training set, $p = 2.4 \times 10^{-2}$; t-test.. (**D**): Boxplot of KRT1 expression differences in the TCGA set,, $p = 4.5 \times 10^{-1}$; t-test. (**E**): Boxplot of IRF6 expression differences in the training cohort, $p = 2.1 \times 10^{-3}$; t-test.. (**F**): Boxplot of IRF6 expression differences in the TCGA set, $p = 7.7 \times 10^{-14}$; t-test.. (**G**:) Boxplot of CDH1 expression differences in the training set, $p = 2.2 \times 10^{-3}$; t-test.. (**H**): Boxplot of CDH1 expression differences in the TCGA set, $P = 1.6 \times 10^{-4}$; t-test.. (**I**): Boxplot of PBX1 expression differences in the training set, $p = 1 \times 10^{-3}$; t-test.. (**J**): Boxplot of PBX1 expression differences in the TCGA set, $p = 6.7 \times 10^{-1}$; t-test.. (**K**): ROC curve from nested cross-validation in the TCGA set, with an AUC of 0.983 (95% CI, 0.942–0.983). (**L**): ROC curve from nested cross-validation in the training set, with an AUC of 0.90 (95% CI, 0.73–1.00).

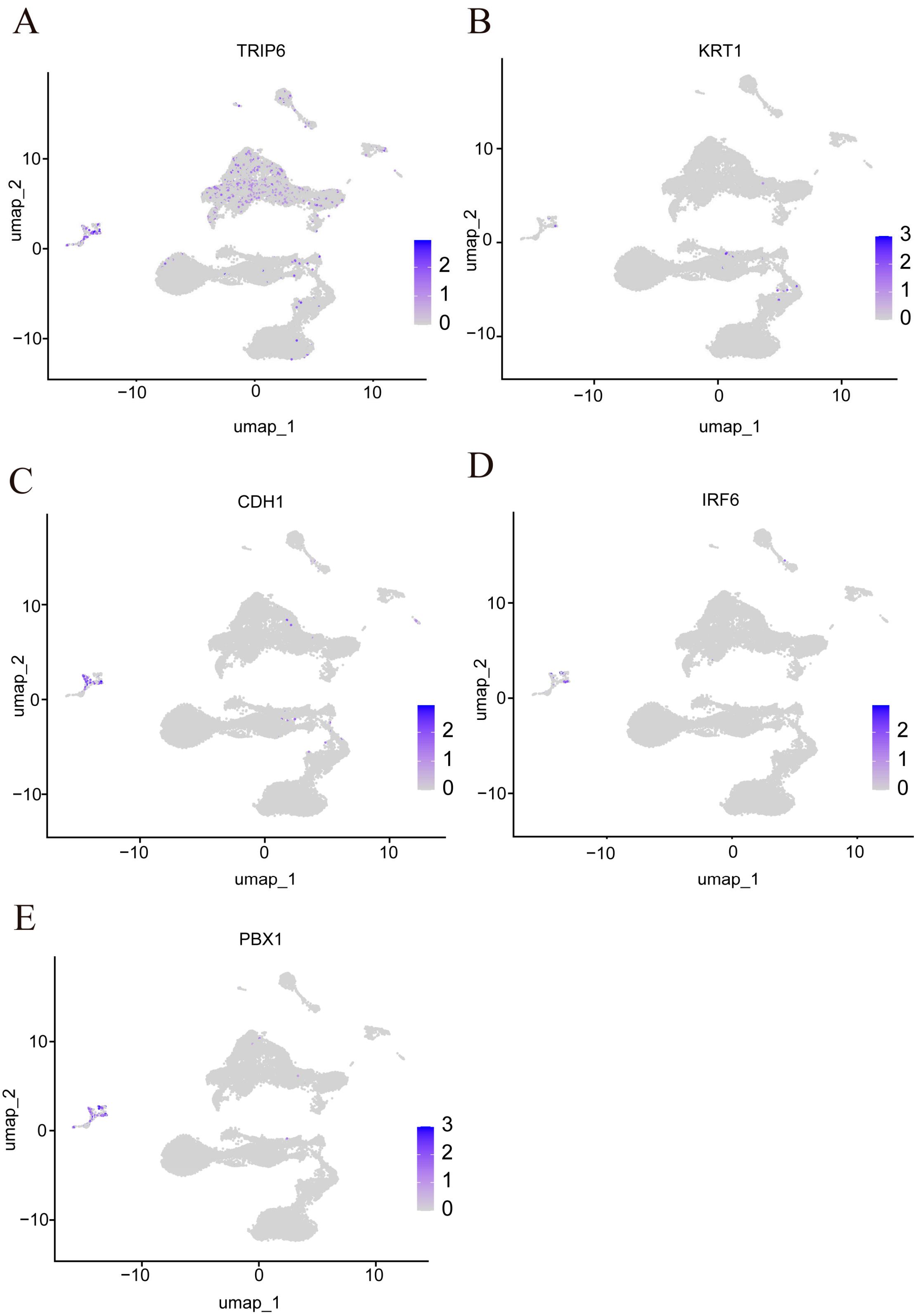

Figure S3: Expression Distribution of Feature Genes in Single-Cell Subpopulations. (**A**): Feature plot showing the distribution of TRIP6 expression across tumor and non-tumor cell subpopulations, with color intensity representing gene expression level. (**B**): Feature plot showing the distribution of KRT1 expression across tumor and non-tumor cell subpopulations, with color intensity representing gene

expression level. (**C**): Feature plot showing the distribution of CDH1 expression across tumor and non-tumor cell subpopulations, with color intensity representing gene expression level. (**D**): Feature plot showing the distribution of IRF6 expression, primarily confined to the tumor subpopulation, with color intensity representing gene expression level. (**E**): Feature plot showing the distribution of PBX1 expression, primarily confined to the tumor subpopulation, with color intensity representing gene expression level.
